# Gynaecological Cancers in India: The Less Heard Perspectives of Healthcare Providers

**DOI:** 10.3390/ijerph20032221

**Published:** 2023-01-26

**Authors:** Kalyani Subbiah, Arima Mishra, Jaya A. R. Dantas

**Affiliations:** 1 Curtin School of Population Health, Curtin University, Perth 6102, Australia; 2 Azim Premji University, Bengaluru 562125, Karnataka, India

**Keywords:** healthcare providers, provider burden, patient provider communication, provider burnout, provider responsibility

## Abstract

There has been mounting evidence on the role of healthcare providers in chronic illnesses such as cancer. The specific complexities in their roles to enable health are less heard. Gynaecological cancers have several undercurrents beyond the obvious. Semi-structured interviews were conducted with healthcare providers in Southern India (*n* = 35) and the data presented in this article were collected as a part of a larger study on the role of communication in the management of gynaecological cancers in India. Thematic analysis of the qualitative data provided information on the providers’ perspectives of gynaecological cancers. Patient numbers, cost, time, cultural norms, context, and institutional constraints in cancer care provision are just some of the factors impacting care provision. Healthcare providers are typically acknowledged for the criticality of their roles in the continuum of care. However, our research suggests that the psychological harm and challenges they themselves may face in providing that care are severely neglected. Through listening to healthcare provider voices, clear solutions emerge to better support the practice of those who are responsible for cancer care.

## 1. Introduction

Severe resource constraints in a health setting like India bring ongoing challenges and ethical dilemmas for healthcare providers responsible for cancer care management [1,2,3]. The primary clinical focus in the care continuum may be on cure and recovery of the patients, but the process and responsibility in the care continuum, especially in gynaecological cancers, requires further investigation and interventions informed by such evidence [4]. Issues such as stigma, difficulties in conducting screening programs, cultural norms in discussing side effects of treatment, and the burden of costs are not separate from the other day-to-day logistics of healthcare provision. They interplay concurrently with the routine of professionals providing care. Outcome-related dimensions such as health promotion for screening, alleviating psychological distress, and preventive practices will be more effectively addressed if this interplay is understood better.

One of the aspects complicating healthcare provision in India is the collusion that exists in cancer care [3]. Families and personal caregivers are more often than not reluctant to disclose the disease status to the women they care for, and healthcare providers are often unwillingly drawn into this conspiracy of silence which the patients themselves may not desire [5]. It places high levels of strain on the healthcare providers who tend to feel divided between what may be in the best emotional interests of the patient and the expectations from family to protect the woman from her own health condition. The cultural norm places the family in a role of significance in such decisions, making situations strenuous, specifically for healthcare providers. Such norms both enable and challenge people depending on the context of care in the cancer continuum. Women are likely to benefit and choose this norm whereas healthcare providers might be in circumstances where they are forced to give the family more importance than they would ideally want to while rendering their services.

Training needs of young aspiring healthcare professionals to address requirements of cancer care continuing is another area full of limitations in India [6]. Similarly, there is insufficient capacity and time for providers to implement screening [7]. Perception and recognition of cancer risks in a timely manner is a key factor in physician training, especially at the primary level of care globally [8]. However, coping and dealing with the uncertainty in a chronic illness is tough on healthcare providers themselves, who have the task of communicating that uncertainty effectively to patients and personal caregivers [9]. Uncertainty permeates many aspects in the care continuum such as prognosis, patients’ and caregivers’ psychological trauma, the efficacy of provider communication and the patients’ cooperation and compliance, and the knowledge and emotional resilience to fight the disease [10]. Awareness and knowledge levels in nursing play a crucial role in cancer care management and are likely to impact the communication with patients that occurs in different stages of screening and care [11,12,13].

In short, there are several distinct dimensions in the cancer care spectrum that span negotiations of treatment, space, interpersonal communication, and availability of services [14]. A chronic illness like cancer also needs to factor in the role and importance of support services, trained paramedical staff, and community health workers who through their liaison and integration can be powerful allies for providers in the health system [15,16,17]. There is an urgent need to find innovative methods of gaining support to overcome resource constraints and training limitations.

Provider communication is important in chronic illnesses such as cancer when the prognosis or information can vary for a patient across the continuum and stage of care. Patients’ understanding of such information is equally vital for all steps forward [18]. Breaking bad news is never easy, and even with the best of experience and intent, the provider may find times when they might have difficulty facing patients who react with deep emotional trauma and distress [19]. Those situations require the support of auxiliary and support services such as social workers and psychologists, which most tertiary facilities in India do not have.

Evidence from an online survey shows that the job satisfaction in cancer care, specifically that of medical oncologists, is poor in most countries of the world [20]. Countries with resource constraints are likely to benefit from collaborative ties with frontline workers who have strong ties to the communities from which patients seek care [21]. The stress and burnout health practitioners deal with in their work is considerable [22,23,24]. There are complex multi-layered issues in the cancer care spectrum for women that are arduous to negotiate.

The literature reviewed aimed to serve a two-pronged purpose. First, the challenges and barriers of healthcare providers are often hidden in the importance they are typically accorded in the health system. Second, the vulnerabilities such challenges create for healthcare providers, in addition, hamper their well-being directly and possibly their responsibilities indirectly. The literature thus highlighted that the lives of healthcare professionals treating cancer patients are difficult and complicated and need to be understood better.

This article aims to examine and highlight the day-to-day complexities in cancer care dealt by healthcare providers in India who manage gynaecological cancers. Through listening to the healthcare provider perspectives, there is scope to better understand the support systems required for this critical group of professionals in the continuum of cancer care.

## 2. Materials and Methods

### 2.1. Study Design

Since the topic is sensitive in nature and required access to women in advanced stages of illness, qualitative methodology was adopted. A case study approach was used since it helped bring the participants’ experiences to the reader and is a method now widely used across disciplines [25]. The data for this study were collected between April 2018 to April 2019 as a part of a larger qualitative and exploratory study focused on gynaecological cancers and the role of health communication in risk reduction in an urban setting in southern India. The geographical location was chosen for the availability of tertiary specialty hospitals offering care for gynaecological cancers, and women who come from different parts of the region and nation.

The viewpoints of the participants and bringing them forth in their own words was feasible. The relativist ontological approach captured the intimately subjective experience that bridges human and individual experience together. As there are multiple individual experiences, so there are multiple realties for the individuals, which was especially true for the participants of this study [26]. We have tried to capture the perspectives and everyday experiences of healthcare providers who are the participants of focus in this paper. A case study approach was the methodology of choice in the study as its main characteristics helped bring the realities of the participants’ experiences to the reader and is a method now widely employed across disciplines [25].

### 2.2. Framework Underpinnig in the Study

The research was underpinned by two conceptual frameworks, the health belief model [27,28] and the theory of communicative rationality [29,30,31], that have aided the unravelling of complex nuances in health decision-making in cancer care.

The communicative rationality approach has shown the factors impacting ongoing decision-making in health among stakeholders (patients, personal caregivers, and healthcare providers), while the health belief model has helped reveal perceptions, triggers, and benefits influencing those decisions. Both these approaches have constructs that highlight the complexity in the consensus, priorities, and approaches among different stakeholders. Communicative rationality reveals the process of agreement among people “living together differently through struggling to make sense together” [29] with sustained initiatives towards consensus in spaces that enable expressing views and respecting another’s.

The health belief model has been applied in health systems for decades since its development to better understand predictors of health behaviour [27,28,32,33,34,35]. The model is relevant to decode the beliefs, barriers, risks, and benefits that influence the perceptions in health behaviour. It also aids the assessment of the self-efficacy of women, their providers, and personal caregivers in the healthcare spectrum. Most importantly, the two models reveal when decisions are made by the participants individually or jointly, and where they disagree or disassociate with each other in health decision-making. In this paper, the perceived risks and susceptibilities are constructs that are particularly relevant, since healthcare providers deal with them at different stages of the disease in the care continuum.

### 2.3. Methods of Data Collection

Semi-structured in-depth interviews were conducted with patients, personal caregivers, and healthcare providers in this study. The main themes for the patient interviews were around identification of disease, treatment and institutional decisions, communication in treatment trajectory, self-efficacy, and health management. Caregiver interviews explored responsibilities in caregiving, norms in caregiving, communication, and information sources; healthcare provider interviews were on experiences with care provision, communication with patients, internal communication, and referral services.

The interview guides were developed drawing on the experience of the researcher in consultation with her supervisors who were experienced in qualitative women’s health research studies in international settings, including India. The guides were translated into Tamil and Kannada by the researcher and two interviewers she has worked with previously when researching women’s health in India. The interviewers were from communities in the same urban setting where the study was conducted.

Consultations were undertaken with a study steering committee comprising health professionals from both clinical and community health settings in the context, on both the identification of study sites and the implementation of the study. Questions were reviewed by the study investigators and translators after the first few interviews to ensure that they adhered to the thematic focus of the study and ensured clarity for study participants.

### 2.4. Study Setting, Recuitment and Ethics

Purposive sampling was used to recruit participants and healthcare providers were recruited from three tertiary hospitals offering advanced cancer care in southern India, cancer support services, and one community-based organisation for cancer care. One tertiary care hospital catered to patients from lower socio income groups, another was a private hospital with a comprehensive specialty centre for oncology services, and the third, a missionary hospital, thatcatered to low- to middle-income groups and had the largest palliative care network for cancer care across the region. Healthcare providers approached included physicians, surgeons, nurses, dieticians and nutritionists, counsellors, psycho-oncologists, patient coordinators, ASHAs (accredited social health activists), social workers, cancer registry staff, organisational heads, and administrative and institutional reception staff. A total of 35 healthcare providers were interviews for the study.

The study received human research approval from the ethics committee of the institution the researcher is affiliated with. Ethical approval was sought and obtained from the Curtin University Human Research Ethics Committee (HRE2017-0739). The study also received permission as per the institutional guidelines of the hospitals and organisations healthcare providers were recruited from. The participants did not receive any reimbursement or token of appreciation for their time as per institutional guidelines. Participants provided informed consent to participate in the study and for the data collected to be used in the research.

Healthcare provider interviews were conducted in their offices and workspaces. If the staff had shared workspaces the interviews were conducted in another confidential room approved by the institution. At times, the healthcare providers from support services were located in the health institutions, but their institutional affiliations were to other organisations. In such cases, the healthcare provider was allowed the opportunity between the consent process and the interview in order to seek approval from their parent organisation. Once the approval was received, the consent process was repeated prior to the actual interview with the provider. If there were work follow-ups, interruptions, or sudden calls that providers had to attend to during the interviews, the interview was paused and only continued after the provider had responded to the situation, even if the interview had to be rescheduled to a later time and date. The interviews with healthcare providers ranged between 30 and 60 min.

All healthcare provider interviews were conducted by the first author. The healthcare providers were in a range of roles in the health institutions that provide tertiary oncology care for women with gynaecological cancers and were directly engaged in providing service or care for women diagnosed with gynaecological malignancies. For their convenience, interviews were scheduled at times that were most convenient for them in the course of their professional responsibilities.

Table 1 describes the range of roles healthcare providers had as well as the number of them designated for each role. It also illustrates the type of institution in cancer care management they were affiliated to. All the providers provided some form of care for gynaecological cancers. If a healthcare provider declined participating in the interview, the contact information of the researcher was left with the provider so that they could contact the researcher any time in the future if they changed their mind. The researcher ensured the healthcare provider could engage in the consent process voluntarily and without the pressure of their responsibilities. All interviews were audio recorded and did not include names or other identifying information such as the participants’ institutions or departments. Participants who declined to have interviews audio recorded during the consent process were not included in the study.

### 2.5. Analysis

The interview data analysis followed several steps including (1) reading of the transcripts and translations to ensure rigour in the data capture; (2) identifying main themes and subthemes in the data; (3) developing a codebook with a list of coded themes; (4) triangulation of data from different categories of respondents in the main study (patients, caregivers and providers) during analysis to enhance validity and identification of themes. The health belief model and the theory of communicative rationality were the theoretical frameworks used for this study. The former has been applied to study the perceptions of barriers and benefits along with risks and susceptibilities and seriousness of conditions that impact health decision-making. The findings of this chapter specifically related to barriers in interpersonal relationships between provider and patients and the flow of information exchange have been understood better with the aid of the health belief model. The need for consensus and collaborative action as evidenced by perspectives from providers has also shown the tenets of collaboration that the communicative rationality framework provides. The latter has been applied to better understand the collaborative cues in such decisions and behaviour. Determination of data saturation was made when repetitive themes were assessed to occur, and for each participant, category interviews were stopped when data saturation occurred (healthcare providers *n* = 35, women patients *n* = 15 and personal caregivers *n* = 10); while other factors such as content validity and quality assurance and rigor of the data were determined with the different study processes including development of a study codebook with joint consultation and review of the questionnaires by the steering committee [36,37,38,39,40].

The interview data required deep engagement with not only what was articulated by the providers openly but dimensions they hinted at or suggested subtly. It was critical to evaluate whether such interpretations were accurate and sensitive and whether they may have been impacted by researcher bias. 

We have followed the basic principles of qualitative research inquiry: the final number of participants followed the principle of saturation of data; efforts were made to purposively include hospitals of different kinds and providers of different kinds who play an important role in cancer care in many ways. Many of these participants were included as participants referred to their roles (e.g., counsellors, psycho-oncologist, patient relations) in their decisions and experiences in the cancer care continuum. 

Since the number does not serve the purpose of statistical generalizability but analytical generalizability, the analysis of the providers interviews took into account the health system context in a relatively resource-poor setting, specific institutional context of care, as well as social and cultural contexts. These help to argue that given similar kinds of contexts, health care providers’ experiences would resonate with the kind of data this study has provided. 

Rigour in the study was ensued through ongoing dialogue and clarifications, evaluation of the major themes and subthemes, refinement of the codebook, and adherence to the research questions. Transcripts were periodically visited to confirm the authenticity of not only the translation but the implications of what was interpreted. To maintain the confidentiality of the participants, none of the quotes from providers are identified by institution, department, or an individual’s role.

## 3. Results

### 3.1. Coping with a Diagnosis Called Cancer

Providers highlighted a difficult dimension of healthcare provision as facing questions for which they have no answers. These questions are about recovery and survival, both of which are often uncertain in cancer care. Fears are expressed by patients about costs, physical pain, duration of treatment, side effects, and death. Costs have specific aspects to them—for example, a particular procedure and what it costs is unknown. Similarly, when what costs will occur is also something that cannot be planned. Providers find the balance between being truthful and simultaneously encouraging about treatment is never easy.

Many healthcare providers said that there are no clear universal guidelines on how a provider can deal with these case-by-case uncertainties with patients and their caregivers. The academic and professional training of the providers does not equip them with these requirements. Each one has their own personal code as a result. The trust of patients and failed treatments imposes a heavy emotional burden.

*Every oncologist goes through this (fatigue)… You are dealing with death and suffering the whole day.*

*When a patient receives a diagnosis of cancer it is almost to her like receiving a penalty or sentence. Whether it is a life sentence or not it is still a sentence*.

Participant ID_Onco_1

*Praying for patients is something that staff often do here. Being there for them and doing something to help. An injection, two words of prayer—we seem to make a difference even with this*.

Participant ID_Palliative_Nursing_2

Providers who have the rare opportunity to find peer support from colleagues are fortunate. It is a safe space for them and reduces emotional burnout and decision-making burden since it is a joint presence and perspective in difficult situations. Providers refer to a deep sense of aloneness in care provision and colleagues can be an antidote to a sense of personal responsibility when treatments fail.

*Learning to understand it is not my fault; it is the nature of the disease and working with a colleague with whom you can counsel [a patient]. This way you can share the responsibility and it really brings down the stress levels… the work used to weigh down on me a lot. Having one more person to share the responsibility helps*.

Participant ID_Onco_1

The providers’ challenges of coping with the diagnosis called cancer emerge from the specific health system and scarcely available mechanisms in it to address these issues. Their experiences further suggest that there are not organized forums/spaces to share such challenges and ways of dealing with such problems.

### 3.2. The Truth That Satys Hidden

Collusion is an area that poses challenge and ethical conflict for healthcare providers. Families repeatedly request them not to share with the patient about her condition. Family plays a vital role in the treatment of a patient, and they tend to lead discussions, make decisions, and manage logistics of treatment, sharing of information, or discontinuance [41]. The family as a result usually decides to protect the patient from the truth about her health and diagnosis. This situation is often in direct conflict with the provider’s need for transparency, especially if the patient wants to know about her status.

The association with the word “cancer” holds high levels of stigma and fear in people’s minds. Providers say that the word cancer is rarely uttered in common parlance in a hospital setting. Rather, they use words such as tumour, biopsy, malignancy, growth, or infection spread to describe the condition to patients. Only when a patient asks her physician directly if she has cancer do they talk about it to her fully or openly. Even in private hospitals where patients can read signboards and readily available printed information, families ask providers not to share the patient’s condition with her. For a provider to accurately gauge what a woman may prefer in this scenario is filled with risk. They speak of instances when a woman agrees to a treatment preference to the provider in front of her family but subsequently reveals in private that her family is coercing her agreement and requests the provider not to reveal to her family about what she has just shared. The provider is placed in an extremely difficult position to negotiate with the family on the patient’s behalf without revealing that the patient herself has stated her real preference in confidence. It sometimes results in families getting angry with providers, refusing to cooperate, and blaming them for any non-cooperation from the patient. Unless handled extremely cautiously, families may discontinue treatment or seek care elsewhere.

*One of the biggest challenges is dealing with differences in what the patient wants and the family wants*.

Participant ID_Psych_1

*Making calls to a patient—one has to be very careful since family may have hidden from her about the disease (from the patient undergoing treatment). The word cancer is usually not mentioned at all unless the family and patient openly mentions it we cannot either… Nursing staff are usually respected a lot in palliative care—often doctors ask for inputs from them on which patients require what kind of specific support…*

Participant ID_Palliative-Nursing_3

Patients and families share confidences with their primary physician provider about personal problems. These tend to be about finances, which may be awkward for a clinician who is both an institutional representative and the primary healthcare provider for a patient. Providers try their best to facilitate available discounts and financial schemes, but coordination is a strain when combined with demanding clinical responsibilities and other patient needs. However, patients are somewhat forced to depend on clinicians for emotional support, because there is a significant gap in the availability of administrative, counselling, and other support services and the numbers of patients who need such support. Many counsellors in such hospitals are volunteers and their time is limited and as equally stretched as that of physicians and surgeons. Relationships with families can be complex and not easy to manage during clinical interactions.

*Higher income groups don’t need much coaxing. The minute they know it is cancer they begin treatment. For women in lower income strata finance is the biggest issue. Social support is a problem—the husband can be very unsupportive… He will have to spend money and she may not be useful at home or may be sexually inactive. We do see women isolated at home, husband doesn’t care, in laws will stigmatize, money is a huge burden along with this… Sometimes they come alone or with their neighbours—without family members. The family may even draw them away from going to the hospital if they confide about problem*.

Participant ID Radiation Onco_1

There are many times when providers know that the patient is well-aware of her illness since her body is giving her cues to her condition. Many patients also keep up the pretense of not knowing for the sake of their family’s peace of mind and do not ask questions. This complicated collusion places a patient in a position of high emotional responsibility for others when she needs to focus on her own wellbeing. It places the providers in an additional uncomfortable role, since they are drawn into and become unwilling participants in family dynamics. However, most of them are motivated by what they discern as a patient’s real apprehensions.

*They will start with complaints and then their worries. You just need to listen to them. Reveal the result in stages. They will know since it is their body. Patient will hide this from family and family will hide it from patient. Patient will act as if she doesn’t know. It can be revealed to the patient slowly. We cannot give false hopes. Oncology can never give false hopes*.

Participant ID_Onco_2

*We shouldn’t be interfering to prove a point. They have to live with their families*.

Participant ID_Pysch_1

There are other instances when psycho-oncologists and counsellors play a pivotal role in family negotiations when there are differing views among the family. A patient can express preferences to providers that she is unable to express to families and directly requests the provider to open negotiations on her behalf. When counsellors intervene in this way, it can result in the families better understanding the patient’s perspective, particularly so when patients do not want to undergo further treatment in very advanced stages and want a peaceful end without intervention.

*Patients have died in the space they want to since we push for internal communication between families*.

Participant ID_Psych_1

Familial tensions can result in a woman being left in a situation where she has no recourse to treatment since she is financially dependent. There are also situations where she is partially or fully abandoned post-diagnosis and has to manage her own health care. Access to short-stay homes is extremely limited and women sometimes have no place to turn to when such situations occur. Women are highly vulnerable when abandoned by a spouse and left with dependent children. Providers then become a primary point of contact and facilitators of aid when this happens. Several providers spoke about a lack of adequate service in this area and stressed that it is one of the most urgent requirements in care management.

Each family reacts differently to the final adverse event of death. Additionally, each institution has its clinical guidelines and protocols on the processes following a patient’s death. Bereavement formalities are mainly handled by the primary physician of the patient along with counselling, where available. Providers expressed the view that while they want to be there for the families through this period, the demands of time and emotion are stressful.

*An oncologist is a friend for life. The day a patient is diagnosed with cancer the oncologist is a permanent person to them. It is a life-long follow-up and association. The door is open even after the loss has happened*.

Participant ID_Onco_1

When a patient is from outside the geographical location of the service, decisions need to be made about transport of the patient’s body. In some cases, providers need guidance about last rites or funeral practices according to the patient’s religion and faith. There are instances where caregivers request that the body not be kept in a hospital mortuary until they can arrange for transport. Such coordination requires empathy, sensitivity, and understanding. Providers also play the role of negotiators between the institution and family for logistics without mitigating the magnitude of the moment for families.

### 3.3. A Limited Luxury Called Time

A recurring theme among the providers was about severe time constraints for cancer care. There are few trained staff in gynaecological cancers. Most women seek care in an advanced stage of the disease, and the complexity of the condition requires extended time to be invested in each patient and her family at diagnosis and at subsequent and different stages of care. However, providers also deal with several patients in their outpatient departments and follow-up stages simultaneously.

*When we handle a new patient with the diagnosis for cancer, definitely no doctor or oncologist can just sit for 5 to 10 min and talk about treatment. We sit for a much longer period with them discussing the disease*.

Participant ID_Onco_3

Providers explained they are not in a profession where they can switch their mobile phones off. A few providers talked about understanding of their families being the reason they are able to handle the pressures of their jobs. They acknowledged that family support was given despite their own inability to invest equally in their personal lives.

Due to low literacy levels among patients, administrative staff spend several hours coordinating documentation and basic aspects of care to the patient and family. Apart from treatment, insurance and financial schemes that are available to lower-income patients also requires extensive paperwork and approval-related follow-up. Linguistic barriers routinely occur since tertiary care is clustered in urban settings, and many patients who are from other parts of the country may not speak the local language.

*Patience is very important in this job. Patients may cause a lot stress but we need to deal with them patiently. This job simply cannot be done otherwise*.

Participant ID_Admin_1

### 3.4. The Silences in Communication

Awareness of risks and the importance of follow-up are two of the most urgent needs in internal communication in a health institution. Providers try to improvise and create their own communication modules and innovate methods to reach their patients more effectively.

*I tell my patients, “Don’t talk about whether you have had your lunch or breakfast. Start talking to one another [other women] about whether you have had a pap smear or breast scan… talk about your health”*.

Participant ID_Gyn_1

*Patient remembers the first day and which doctor explained the treatment the first day. Everything matters on that first day. If you can create confidence, then they will be there for the treatment long-term and they usually look for you [whenever they visit hospital]*.

Participant ID_Onco_4

Interpersonal communication and health information for patients should be customised significantly by providers based on individual understanding, needs, and receptivity of patients and families. Lack of context-specific data increases this load on providers.

*Unfortunately, in India we don’t have data, we don’t have statistics on outcomes of disease or treatment; we follow American or European guidelines [in practice].*

*Most of the literature is from the West. The tumours and our patients are both different; race, nutrition, finance, social codes are all also different*.

Participant ID_Onco_1

Patient referrals from doctors is essential for counselling support to be provided in a timely manner but referrals are often made based on symptoms or complaints from a patient. Counsellors discuss the needs their colleagues themselves have for counselling support. Palliative care providers speak about the problems with their services being the last point of referral when all other options are closed. The unspoken interpretation that palliative care is end-of-life care hampers patient openness to seeking it.

A patient is not referred immediately for counselling support since emotional trauma and physiological symptoms are considered natural and normal during the treatment period. Hospitals focus on patient diagnosis, care and treatment plans and there are no avenues to talk about difficulties in treatment due to constraints of time, confidentiality and limited institutional resources. There is also the silent expectation for the providers to be strong, courageous and confident under all circumstances.

### 3.5. Grief, Fear and Mortality

Gynaecological cancer care is interconnected with apprehensions about mortality. Most patients and families respond to a diagnosis as the commencement of a ticking clock against them regardless of the stage of the disease. Providers acknowledge that even where there are good prognosis and treatment outcomes, their experience suggests that one can never guarantee complete cure. Women are additionally concerned about the impact of the disease and treatment side effects on their roles as wives and mothers. Patients are willing to take high risks and discontinue treatment in order to preserve their reproductive organs, especially younger women yet to have children. The negotiations providers have with patients and families are extremely sensitive, since treatment side effects could have severe implications on the marital life of a woman. Sometimes these decisions become a complex weighing of the risk to a woman’s life vis-à-vis the risk to her sexual activity, marriage, and acceptance by those closest to her. Providers must deal with such issues with tremendous thought since a recommendation for treatment despite opposition from family could elicit strong resistance and may even push a woman or her family to discontinue treatment.

*I get very upset when surgeons or oncologists say we will “cure” cancer… we are fooling ourselves and fooling everybody else*.

Participant ID_Onco_2

Mortality due to gynaecological cancers is high in low- and middle-income countries due to poor screening efforts, sociodemographic factors, and access to advanced radiotherapy facilities [42]. India recorded 97,000 cases in terms of the global burden of cervical cancer and 60,000 deaths in 2018 [43]. Providers are the primary people to manage grief from the patients and the family at all stages of treatment. Grief could also manifest itself in a variety of other ways like anger, fear, depression, silence, and non-cooperation towards the providers. The treatment for gynaecological cancers in advanced stages is long and typically patients and their caregivers are in the hospital for at least 6 to 8 months. Both families and providers face ongoing uncertainties of prognosis. An ethical dimension enters the situation when financial instability occurs. Families seek answers on whether or not they should continue spending for treatment if there are no positive outcomes. Providers deal with both harsh realities: patients and families who simply cannot accept failed treatments with no options left, and those that insist on continuing even when the prognosis is very poor and they cannot afford the costs.

By the time a patient reaches palliative care, pain management and acceptance from the patient herself become crucial. Providers are called upon to manage pain, be an emotional confidant, or just pray, depending on the requirement for the day and time. The relationship becomes very personal for the family, and providers in this study discussed the strenuous responsibility of maintaining their professional equanimity and emotional distance as needed. Delayed referrals accentuate these issues a great deal.

*It is important not to come in for support when death is right there*.

Participant ID_Psych_1

In home-based palliative care, providers sometimes see their patients being poorly treated, neglected, or verbally abused by families that are too exhausted themselves and are no longer able to care for the patients. Basic access to hygiene that is fundamental for preventing infections or relapse may be missing in resource-limited homes. However, any intervention or feedback may adversely impact the relationships for the patient in the household and worsen her condition.

*In the hospital it can be mapped, but once you enter a village several issues need to be understood. We have to follow them till death*.

Participant ID_Palcare_2

### 3.6. Treatment Failure, Burnout and Self-Doubt

Losing a patient is personal for each healthcare provider though the way they cope, address, and respond to it may differ. Many of them talk about the helplessness of failure and the guilt of not having met the main responsibility a patient and family trusts them with—cure and life extension. While they rationally accept variables determining outcomes, there is a deep sense of personal guilt providers carry.

*I get very involved with patients… my mentors over the years have had a lot of influence on the way I deal with patients*.

Participant ID_Onco_2

*It is emotionally draining. We see death. We see a lot of suffering. Not just physical but psychological, spiritual distress and we see social distress*.

Participant ID_Palcare_1

Patients and families often ask providers to make most decisions on their behalf since they rely on their expertise and experience. While on the one hand this creates a veneer of authority and confidence in healthcare provision, below the surface are the lonely pressures of decision-making that could go very wrong. Some providers try to lessen these pressures by not getting involved with the patients’ and families’ emotional trauma. Emotional burnout early in their careers is something they learn from and try to avoid repeating in their careers. The constant need for reassurance places a huge weight on the already overworked doctors, nursing staff, and counsellors. Many acknowledge that though they try, long-term associations make it impossible to completely disassociate from the personal reactions, ongoing fear, and anguish of patients and families.

*If I try to manage caregiver burnout, we ourselves will also have burnout so I have stopped doing that*.

Participant ID_Onco_5

As there is a shortage of trained nurses and staff in the specialty of gynaecological cancers, staff are required to increase their responsibilities. Providers express their awareness of burnout, stress, and unmanageable workloads even though a smile from their patients makes everything worthwhile. Experienced providers were seriously concerned about apathy among young providers because of a lack of training and the increasing commercialization of medicine. Empathy and compassion, according to many providers, are as important as the medical training received for cancer care.

*They will hear half, do half, understand half and patient will be treated half or zero [about trainings conducted with junior colleagues]*.

Participant ID_Nutri_1

Most of the providers acknowledged intermittent or an ongoing sense of burnout that they experience in their work. Their methods of coping were different and practiced by trial and error at an individual level.

*I just shut down and not talk to anyone, compassion fatigue is high in the work and it is important to de-stress… Don’t hide the stress; face it and acknowledge it and be mindful*.

Participant ID_Psych_1

*I have not got to the stage of figuring out how to de-stress myself [when a patient has poor outcomes]. I have noticed one way that helps me is to move on to the next patient*.

Participant ID_Onco_2

### 3.7. A Space Called Spirituality

Where all hope is lost, some providers say that spirituality tends to provide the maximum solace and comfort to people. Other providers expressed how they themselves faced existential crises that question their faith due to the pain and loss they deal with routinely in their work. Healthcare providers in palliative care are particularly articulate about the role spirituality plays in their day-to-day lives. Several of them mentioned faith as a source of strength to cope with pain, suffering, and loss.

*I got more compassion [for patients] after I began actually experiencing God’s love. I started feeling peace*.

Participant ID_Palcare_3

*Some oncologists put their reliance in God. I have actually questioned my belief in God after being in oncology. If we pray or do something does someone really listen to all this? I have had my own spiritual challenges when dealing with patients with cancer*.

Participant ID_Onco_1

Nursing staff also explained that spirituality teaches them to bear and be respectful of high degrees of physical suffering their patients and families undergo. The word “healing” was used by many staff when speaking of the nursing care that patients need, and that they coped with this responsibility only with the aid of their personal spiritual faith. Some providers said spirituality gives them solace and meaning especially when they reach internal spaces of hopelessness.

## 4. Discussion

The research has shown that healthcare providers are adversely impacted by the challenges of time constraints, limited support services, and emotional burnout. This is consistent with global literature that highlights the importance of responding to provider perspective and burnout in chronic illnesses and end of life care [44,45]. The literature highlights how support services and models of care-integrated services to assist healthcare provision should be evaluated thoroughly to understand if their efficacy is context-specific and that the services need to be tailored appropriately with the views of key professionals [46,47,48]. Being a qualitative study, the depth of information, providing voice, transferability of data, and data saturation were important considerations. While individual providers talk about different dimensions of support, the recurrent theme among them is the hardship of managing their responsibilities alone while handling varying demands in care management. They are united in the view that cancer care management is extremely challenging, emotionally arduous, and fraught with guilt, burnout, loneliness, and self-blame. The social and cultural complexities in care provision require collaboration between providers, institutions, patients, families, and allied services.

Evidence suggests that major illnesses have complex expectations in health-seeking behaviours, and these negotiations about illness and wellness impact relationships between providers and patients [49,50]. The research shows that these relationships are further aggravated and distressed due to a dearth of resources and a lack of open forums to discuss these issues. There is a general consensus among providers that even when they run out of all options and have no assistance, they would never choose to give up on a patient.

The frameworks of communicative rationality and the health belief model underpin the findings that there are several barriers both for their work with patients and their own inner wellbeing. For interventions to be of relevance, the interplays of perceptions of patients, personal caregivers, and institutions towards cancer care need to be fully deciphered since they directly affect the context of providers. The decisions that providers make in cancer care are surrounded by uncertainties that span a range of issues such as patient communication, prognosis, role of personal caregivers, and institutional guidelines [51].

The health institution is still the central variable of care provision and experience for a patient and her family while the provider is in many ways the epicentre in that decision-making process. While this perspective might seem organizing towards patients and their families, it is the harsh reality in the care spectrum where these groups engage with each other. The provider being an important point person is not about placing him or her higher in the hierarchy. It is about organizing the power they wield just by the nature of their responsibilities. This inference certainly does not imply that this is ideal or desirable in the health spectrum, simply that this is the reality in a context like India for many women. The interventions or support services that are activated to enhance their work should enable providers in the health system, rather than act as external problem solvers. For this, a thorough and well-informed understanding of providers’ needs is essential.

An area where allied services can offer definitive support in the existing scenario is in increased counselling support for patients and in organizing patient centric events in the hospital. This can also be combined with health information both in the health institutions and in local communities. Clinicians reiterate that the persons or groups involved with such engagement need to be trustworthy and credible. Appropriate services that institutions endorse and collaborate on may be the solution. An objective review by individual institutions on how joint or collaborative care can be provided by multiple providers in patient interactions and treatment follow-ups, especially the challenging ones, is essential.

The other area that requires investment is counselling and professional support for providers themselves. An internal referral system that makes periodic counselling mandatory for all providers in cancer care may be an approach to consider. Alternatively, easily accessible and informal confidential counselling support outside the institution or through a reliable helpline may also be an option that will help providers significantly.

A key aspect such interventions and support services need to recognise is that they cannot add to the existing workloads of healthcare providers. The conflicting emotions and apprehensions that a provider faces in disease management are rarely shared. There is the requirement for the health system to recognise this and make available supports that anticipate providers’ needs before they themselves do. Neutral and enabling spaces where providers can just sit and recharge or heal by themselves, and sometimes with each other, may be another dimension of physical and psychological benefit. Institutional boards for case management can also convene sessions to discuss provider burnout, stress, and other individual or collective concerns. The silences that surround provider needs must be broken without fail from within institutions.

The recommendations of providers are clear and categorical. Support from colleagues to handle difficult conversations including treatment failures and end of life care is necessary. Time allocation in clinical care can be improved with well-functioning support services to handle the secondary needs of patients such as administrative and funding support, health information modules, patient counselling, and personal caregiver services. Internal coordination between clinical care and palliative care within institutions will also aid patient care far better. Work in the community in the form of preventive care support and screening can benefit more with the involvement of community-based organisations. The need for more holistic education about patient care is imperative. It can be better synchronised in the current academic curriculum so that there is necessary sensitisation and capacity building before the careers of health providers commence. The study findings strongly suggest that the neglect of these key professionals can aggravate challenges severely for them and those they care for long-term.

## 5. Conclusions

With this research, we contribute to alleviating some gaps in the literature about the challenges healthcare providers face in India in the management of gynaecological cancers, especially in relation to the conflicts within their professional responsibilities vis-à-vis their own wellbeing. Providers of care in gynaecological cancers have major unmet needs. Their perspectives reveal how the gaps in allied personnel support, information dissemination, and confidential emotional support affect their responsibilities. These individuals bear tremendous responsibilities to help and heal others. It is imperative to hear the perspectives of healthcare providers managing gynaecological cancers in India, along with a focused intent to alleviate their problems.

## Figures and Tables

**Table 1:** Institutions and Roles of Healthcare Providers.

Type of Institution *	Role of Healthcare Provider	Numbers
Tertiary subsidised hospital	Head: Oncology Oncologist: Radiology Oncologist: Surgery Nurse: Oncology outpatient Nurse: Oncology ward Patient relations: Front desk Counsellor Dietician Head: Cancer registry	1 4 2 2 2 2 2 1 1
Tertiary private hospital	Head: Gynaecological Service Line Manager Patient Relations: Front desk Psycho-oncologist	1 1 1 1
Tertiary missionary hospital	Palliative care: Head	1
	Palliative care: Home care physician	2
	Palliative care: Nurse	4
	Oncologist: Radiology	1
	Oncologist: Surgery	1
	Counsellor	1
Support services	Senior counsellor & founder	1
Community based organization	Founder & director Medical director Community health worker	1 1 1
		35

* There was no concordance between the types of hospitals and the providers. One, as a teaching hospital had more clinicians while another had more auxiliary staff. Only the missionary hospital had a palliative care department with staff specific to that department. In many cases, the counsellors served in other hospitals parallel to their responsibilities in these hospitals.

## Data Availability

The data presented in this study are available on request from the corresponding author.
